# Cerebral Venous Thrombosis Mimicking a Discrete Brain Mass: A Case Report and Literature Review

**DOI:** 10.1155/2018/5862912

**Published:** 2018-05-17

**Authors:** Dana DiRenzo, Zsuzsanna H. McMahan, Naman S. Desai, Rebecca Manno, Michelle Petri

**Affiliations:** ^1^Division of Rheumatology, Johns Hopkins University School of Medicine, 5200 Eastern Avenue, MFL Building, Center Tower, Suite 4100, Baltimore, MD 21224, USA; ^2^Division of Neuroradiology, The Russell H. Morgan Department of Radiology and Radiologic Science, The Johns Hopkins Hospital, Phipps B-100, 600 North Wolfe Street, Baltimore, MD 21287, USA

## Abstract

The differential diagnosis for a focal brain lesion in a patient with systemic lupus erythematosus (SLE) is broad and includes infection, malignancy, and vascular and inflammatory etiologies. One rarely considered vascular pathology is cerebral venous thrombosis (CVT), which is often associated with a delay in diagnosis because of variable presentation and rare incidence. We present the case of a young woman with a new discrete brain lesion that appeared in the context of highly active SLE and was ultimately diagnosed with a CVT. We provide a literature review for diagnosis and management of cerebral venous thrombosis, a potentially serious complication of untreated systemic lupus erythematosus.

## 1. Case

A 28-year-old female with a history of lupus developed chest pain, altered mental status, and fever over a seven-day period. Her lupus was characterized by high titer anti-nuclear antibody (1 : 640), anti-Sm antibodies, anti-RNP antibodies, hypocomplementemia, oral ulcers, and alopecia. Supportive serologies included anti-Ro and anti-La antibodies; anti-cardiolipin and anti-*β*2 glycoprotein antibodies were negative. Electrocardiogram on admission was consistent with pericarditis, and inpatient treatment was recommended. However, the patient eloped only to return several hours later complaining of headache. Due to progressively odd behavior, magnetic resonance imaging (MRI) was obtained, which showed a new brain lesion in the left dorsal insular cortex. She was transferred to our large tertiary care center for further evaluation and management.

On initial physical exam, she had alopecia and oral ulcers. Her neurologic exam was notable for a tangential thought process, slightly slower fine finger movements on the right compared to the left and slightly brisker reflexes of the lower extremities compared to the upper extremities. Her upper and lower extremity strength and sensation, cranial nerves (II–XII), and gait were normal. Initial lab work showed a normocytic anemia (hemoglobin 9.3 g/dL) with normal white count and platelets. Renal function was normal (creatinine 0.75 mg/dL) but with nephrotic-range proteinuria (spot urine protein/creatinine ratio 9.8 g/day). Complements were low (C3 47 mg/dL and C4 4 mg/dL), and inflammatory markers were elevated (erythrocyte sedimentation rate 94 mm/hr; CRP 1.18 mg/dL). She also had significant hypergammaglobulinemia at 3000 mg/dl (normal 382–929 mg/dl). Large pleural and pericardial effusions were found on body CT imaging.

**Figure 1:**
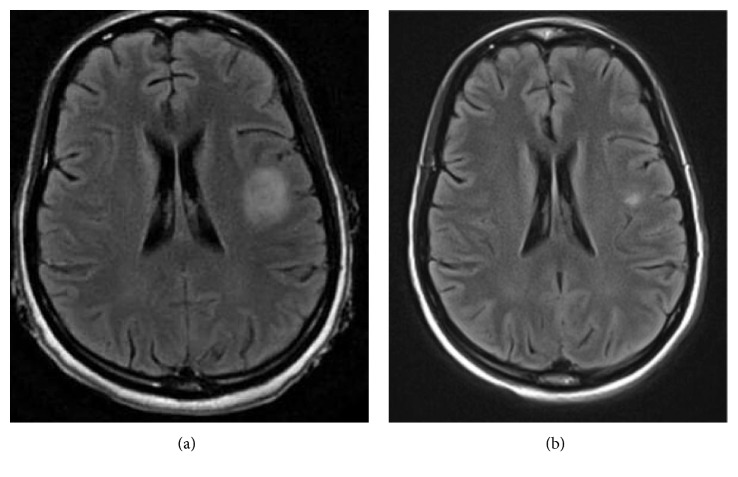
(a) MRI brain with and without contrast on admission (T2-FLAIR image) demonstrating focal mass in the dorsal left insular cortex and subcortical white matter. There is surrounding edema with little mass effect. (b) MRI brain at a 10-day interval follow-up demonstrating marked improvement in the dorsal left insular brain lesion (T2-FLAIR image).

Brain MRI with and without contrast showed a focal mass with surrounding edema but no mass effect and reduced peripheral T2-weighted signal (Figure 1(a)). Weak postgadolinium T1-weighted signal enhancement was present. Biopsy of the lesion was considered but determined to be too risky due to damage to critical surrounding brain structures. A lumbar puncture revealed normal white blood cell count and mildly elevated protein levels at 56 mg/dl (normal 15–45 mg/dl). CSF cultures were negative. PET-CT scanning showed low FDG uptake at the site of the brain lesion compared to surrounding brain parenchyma and diffuse lymphadenopathy with a maximum of 3.0 standard uptake values (SUVs) in the right axilla. A fine-needle aspiration of a right axillary lymph node showed reactive lymphadenopathy with negative flow cytometry and culture. Due to concern for brain abscess, broad-spectrum antibiotics and acyclovir were started. Corticosteroids were not started at this time, despite evidence for some lupus activity with serositis because of concern for brain abscess.

Repeat MRI scan of the brain with and without contrast and magnetic resonance venogram (MRV) 10 days after the initial imaging showed significant improvement in the “mass” with patent dural venous sinuses and cortical veins (Figure 1(b)). Multiple blood and urine cultures, including fungal cultures and cultures for *Mycobacterium*, remained negative.

In the following days, the pericardial and pleural effusions grew to a critical size requiring urgent pericardiocentesis and thoracentesis. Cultures from this fluid were also negative. A renal biopsy to characterize suspected lupus nephritis was scheduled but delayed due to recent ingestion of nonsteroidal anti-inflammatory drugs causing an increased bleeding risk.

**Figure 2:**
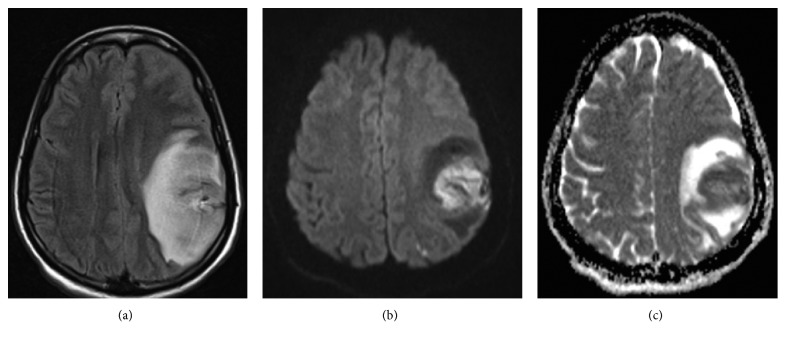
Axial FLAIR (a) representative image demonstrates large area of FLAIR hyperintensity involving the left frontoparietal region representing edema with mass effect, new/significantly increased compared to the prior study. There is associated restricted diffusion (as evidenced by DWI hyperintensity (b) and ADC hypointensity (c)) compatible with evolving acute infarct.

**Figure 3:**
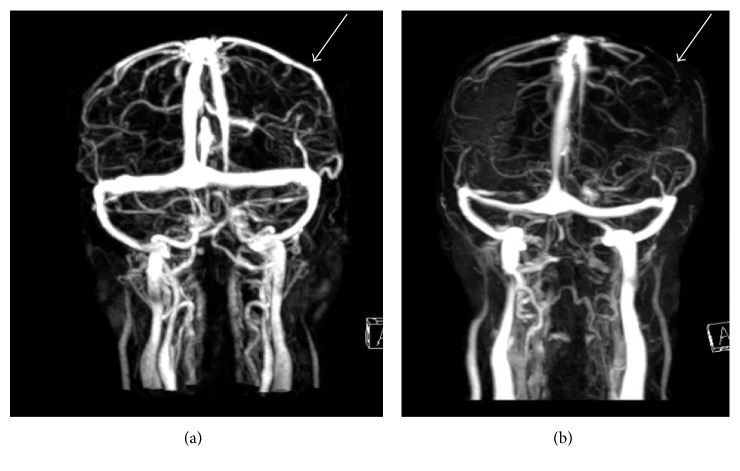
Contrast-enhanced MRV of the brain is performed on day 10 (a) and day 16 (b). There is patent left vein of Trolard on day 10 with good collateral flow involving the left frontoparietal region. However, on day 16, there is thrombosis of the left vein of Trolard with slight decrease in collateralization within the left frontoparietal region, compatible with venous thrombosis and hemorrhagic venous infarct.

**Figure 4:**
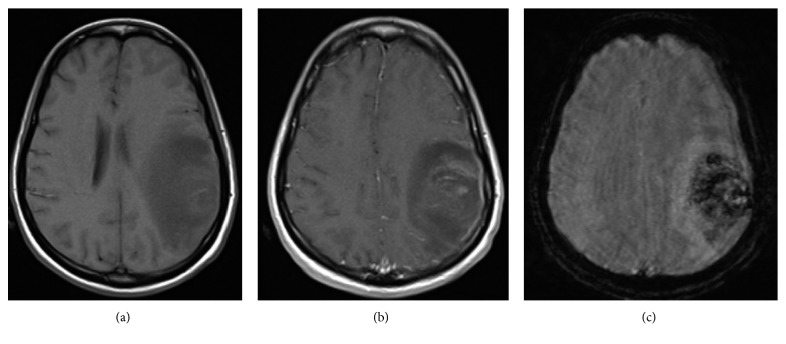
On day 16, corresponding axial T1 precontrast (a) and postcontrast image (b) does not demonstrate rim enhancement or a fluid cavity to suggest an abscess. Additionally, except for some thin patchy postcontrast enhancement in the region of the vasogenic edema (which could represent some venous engorgement secondary to mass effect), no mass-like postcontrast enhancement is noted to suggest an enhancing tumor. (c) Corresponding susceptibility-weighted image demonstrates ill-defined areas of hemosiderin deposition in the left frontoparietal region representing areas of hemorrhage.

On day 16 of admission, she developed acute left facial droop with right arm weakness. A D-dimer was elevated to 17.02 mg/L (normal 0.19–0.52 mg/L). A large hemorrhagic venous infarct in the left frontal and parietal lobes (Figure 2) with abrupt cutoff of the vein of Trolard and superficial draining veins was evident on MRV with a 7 mm midline shift (Figure 3). Corresponding axial T1 pre- and postcontrast imaging was not suggestive of an underlying enhancing tumor but rather hemorrhage with hemosiderin deposition in the left frontoparietal region on susceptibility weighted imaging (Figure 4). IV solumedrol and mycophenolate mofetil were added for treatment of presumed lupus nephritis and serositis in the setting of a negative infectious workup. She was transitioned to warfarin for continued anticoagulation, and the neurologic deficits improved over the next several weeks.

## 2. Assessment

Hemorrhagic conversion of an evolving CVT that mimicked a discrete brain mass was diagnosed. This was supported by rapid resolution of initial MRI edema (without intervention), lack of FDG uptake of lesion on PET, and clear MRV infarct once the clot propagated to a significant size.

## 3. Discussion: Cerebral Venous Thrombosis

CVT is a rare vascular phenomenon accounting for 0.5% of strokes with the average tertiary care center seeing about 5 to 8 cases per year [1, 2]. In the only large, single-center, retrospective review of patients with lupus, CVT occurred in 17 out of 4,747 patients over a 13-year period [3]. In contrast, Behcet's disease, another autoimmune disorder, carries a greater risk of CVT with 64 out of 820 patients experiencing an event in one retrospective cohort [4].

The underlying cause of CVT is varied. CVT is generally secondary to a hypercoagulable state such as malignancy, oral contraceptive use, pregnancy and puerperium, venous stasis, or mechanical obstruction [5]. Acquired and inherited hypercoagulability accounts for a large percentage of CVTs including systemic inflammatory diseases, nephrotic syndrome, antiphospholipid syndrome, protein C and S deficiency, factor V Leiden, and methylene tetrahydrofolate reductase mutations, among others [6, 7]. CVT has infrequently been reported as the presenting feature of SLE with risk factors including thrombocytopenia, a high disease activity index, and antiphospholipid antibodies [3, 8].

One of the challenges in diagnosing CVT is the nonspecific signs and symptoms which can mimic many other intracranial pathologies. In two retrospective analyses of patients with cerebral venous sinus thrombosis, the majority of patients complained of headaches, either of acute or subacute onset, as initial presenting symptoms [7, 9]. In one retrospective study, headache was the only symptom in 17 out of 123 patients with CVT [10]. Intracranial hypertension, seizures, encephalopathy, and focal neurologic deficits have all been reported as presenting symptoms [11]. This heterogeneity in presentation is the common reason for delay in diagnosis, which is on average 10 days after initial presentation [12].

The appearance of CVT on imaging can mimic tumors or focal infections, such as occurred with our patient [13]. T2-weighted MRI with MR venography is accepted as the most sensitive diagnostic tool for CVT [14]. Vasogenic edema is more readily apparent on MRI T2 and fluid-attenuated inversion recovery (FLAIR) images compared to CT with areas of increased signal [15]. In our patient, the initial mass-like findings with edema likely represented an early venous infarct. A second more significant thrombotic insult increased clot burden causing propagation of the infarct leading to focal neurologic deficits and obvious MRV findings (Figure 3) [16]. This is also consistent with the lack of lesion's FDG uptake on the PET scan, whereas a focal infection or malignancy almost universally is FDG avid [17]. The lesion did not demonstrate a fluid cavity or rim enhancement to suggest an abscess. It should be noted that mass lesions are generally not associated with lupus in the absence of infection, malignancy, or vascular insult, such as CVT. New diagnostic techniques, specifically the MRI 3D T1 SPACE, show improved visualization of subacute CVTs and may improve diagnostic accuracy and delay in diagnosis in the future [18, 19].

The mainstay of therapy for CVT is anticoagulation with treatment of the underlying disorder, if applicable [20]. Unfractionated heparin versus low molecular weight heparin are initial agents of choice with acceptable safety profile and nonsignificant reduction in mortality and dependency according to a Cochrane review [21]. Decompressive hemicraniectomy or endovascular intervention may be necessary in the case of progressive neurologic symptoms, coma, or increasing intracranial hypertension [22, 23]. Our patient received glucocorticoids to alleviate cerebral edema, causing a midline shift as noted on the MRI. According to the American Stroke Association, duration of therapy is determined by provocation [14]. If a CVT is provoked secondary to transient risk factors (infection, malignancy, pregnancy, etc.), vitamin K antagonists should be continued for 3–6 months with a target INR of 2–3. If a CVT is determined to be unprovoked, as could be the case in inherited thrombophilia and related disorders, anticoagulation with vitamin K antagonists should be continued lifelong with a target INR of 2-3. Mycophenolate mofetil was chosen as the first-line agent for the treatment of our patient's renal disease and serositis given her race and desire to preserve fertility [24]. Nephrotic syndrome and high disease activity were felt to be the primary drivers of our patient's thrombotic event.

Long-term outcomes for patients who have suffered from a CVT are more favorable than arterial infarcts with full neurologic recovery expected in most patients [25].

## 4. Conclusion

CVT is a rare and potentially serious vascular complication that should be considered in patients with highly active SLE, especially those with nephrotic syndrome secondary to untreated lupus nephritis. Prompt treatment of the CVT typically has favorable patient outcomes. Other risk factors for CVT in patients with SLE include thrombocytopenia and antiphospholipid antibodies. If neurologic abnormalities are present, an MRV should be considered early in the diagnostic algorithm, as this is the most sensitive modality for screening of CVT.
